# Effectiveness of Watching a Kumagai Method Video for Long-Nipple Bottle-Feeding for Children with Cleft Lip and Palate: A Pilot Experimental Before–After Trial Study

**DOI:** 10.3390/children11111358

**Published:** 2024-11-08

**Authors:** Shingo Ueki, Yukari Kumagai, Yumi Hirai, Eri Nagatomo, Shoko Miyauchi, Takuro Inoue, Qi An, Eri Tashiro, Junko Miyata

**Affiliations:** 1 Department of Health Sciences, Faculty of Medical Sciences, Kyushu University, Fukuoka 812-8582, Japan; 2 Department of Nursing, Osaka University Dental Hospital, Osaka 565-0871, Japan; 3 Department of Advanced Information Technology, Faculty of Information Science and Electrical Engineering, Kyushu University, Fukuoka 819-0395, Japan; 4 Department of Informatics, Graduate School of Information Science and Electrical Engineering, Kyushu University, Fukuoka 819-0395, Japan; 5 Department of Human and Engineered Environmental Studies, Graduate School of Frontier Sciences, The University of Tokyo, Chiba 277-8563, Japan; 6 Department of Pediatric Surgery, Graduate School of Medical Sciences, Kyushu University, Fukuoka 812-8582, Japan

**Keywords:** bottle-feeding, children, cleft lip, cleft palate, instructional video

## Abstract

Aim: This study aimed to determine whether the Kumagai method could be followed by watching an instructional video and to compare the feeding actions of specialists and the general population. Materials and Methods: Eleven adults from diverse backgrounds were recruited and asked to feed a baby doll using a bottle feeder with a long nipple, and their actions were recorded. Then, they watched a 2.5-min instructional video of the Kumagai method and were asked to bottle-feed again. Video recordings of the participants were used to evaluate whether their actions followed the Kumagai method. Differences in the feeding actions of the specialists and participants were determined from still images in the videos. The McNemar test was used to analyze the differences between the participants’ actions before and after. The Kruskal–Wallis test was also used to determine differences between the actions of participants and specialists. Results: Feeding movements significantly improved after watching the video. Participants’ actions, which differed significantly from those of the specialists, were evident in “the angle of the nipple at the moment the nipple was brought close to the lips” and “the angle change of the nipple from the lip to the mouth”. Conclusions: Watching our short instructional video was effective in improving the technique of the Kumagai method; however, it is insufficient for feeding a real child with cleft lip and palate. Including the rationale of each action in the video and adding direct instruction as a supplement is required.

## 1. Introduction

Cleft lip and palate (CLP) is a congenital disorder caused by defective fusion of the lips and palate during the fetal stage [1]. It is the most well-known oral and maxillofacial malformation. Globally, its prevalence per 1000 live births is 0.45 [2], and in Japan, it is much higher, at 1.17 [3]. Infants born with CLP face many challenges, including feeding difficulties, dysarthria, dental abnormalities, and issues related to facial aesthetics [4]. Moreover, their parents experience difficulties in raising them, such as in deciding when to explain the disease to their child or how to respond if the child is bullied [5]. Therefore, CLP requires multidisciplinary management involving healthcare professionals from various disciplines, such as pediatric surgery, dentistry, speech therapy, and nursing [6]. Feeding difficulties are the first and most common challenge faced by children with CLP after birth. The feeding mechanism involves the sucking of the nipple accompanied by a wave-like peristaltic movement of the tongue [7], which creates a vacuum when the tongue is at its lowest position [8]. These mechanisms are enabled by the palate. However, infants with CLP are prone to feeding difficulties due to their inability to form negative pressure to effectively suck [9].

Feeding tools such as squeeze bottles, syringes, and paladai that do not require sucking are effective in improving feeding volume in children with CLP; however, scholars and practitioners disagree about the most appropriate feeding method for these children [1]. Cup feeding is another method used in many countries. However, evidence of its benefits is lacking, and the method can potentially increase the length of hospital stay [10]. Feeding is significant not only for nutritional intake but also for the development of oral motor function. If oral movements during feeding are not maintained under proper conditions, harmful oral habits may develop, leading to disorders such as changes in palate morphology, malocclusion, and gingival changes [11]. In addition, ulcers may form on the mucosa if the nipple strikes the nasal septum because of the striking motion of the nipple with the tongue [12]. Lip modification and palatoplasty are typically performed in infants with CLP before the age of one year, which improves nutritional status post-surgery. Postoperative feeding should place minimal stress on the lip or palate to ensure rest for the wound.

A bottle feeder with a long nipple, called the Long Nipple, has been developed to help feed infants with CLP smoothly and without strain. The Long Nipple is thin, long, and made of soft silicone rubber. Unlike ordinary nipples, which require a child to suck, this is designed to be held between the fingers to eject milk [13]. However, it is difficult to use. Even a slight tilt or bending of the nipple could easily eject milk. Therefore, incorrect feeding techniques may result in infants refusing to feed. By placing the nipple in the proper tongue position, infants learn to use their tongue, similar to a child without CLP when drinking breast milk. Ms. Kumagai, the director of nursing at a university hospital in Japan, has spent years establishing the technique for feeding infants using the Long Nipple. The technique has been named the Kumagai method. Its technical procedure has been recently revealed [13]. According to one Japanese report, the time taken by an infant to consume 40 mL of milk could be reduced from 60 to 10 min with the Kumagai method [14]. Even children with CLP who have difficulty feeding from a regular bottle could expect to improve their feeding volume and nutritional status using the Kumagai method. Therefore, it is necessary for more people to know, learn, and master this method.

The method is considered highly reproducible, as many parents of children with CLP could learn and master this method with enthusiastic teaching by an expert nurse. However, this method has been shown to them directly by face-to-face instruction from expert nurses and has not yet become widespread, with few facilities in Japan currently using the Long Nipple [15]. We showcased videos on YouTube in December 2021 explaining the basic techniques to increase its adoption and effective use and made them available to the general public. The video was mainly made for the caregivers of children with CLP and so that it would be easily understandable by the general population. YouTube videos have shown significant effectiveness as a means of providing health-related information [16]. It is necessary to confirm the effectiveness of our video and investigate the possibility of further educational interventions. Therefore, this study aimed to determine the possibility of the effectiveness of instructional videos in helping a wider population master the Long Nipple method and to examine the differences in feeding movements between Kumagai method specialists and the intended viewers.

## 2. Materials and Methods

### 2.1. Design

This pilot experimental study adopted a before–after trial design to determine whether an instructional video of the Kumagai method could help viewers acquire the techniques. Standards for Quality Improvement Reporting Excellence in Education (SQUIRE) [17] were followed to guide reporting in this study.

### 2.2. Participants

Data were collected from September to November 2022. Through convenience sampling, we recruited 11 participants, comprising 4 healthcare providers (2 midwives and 2 pediatric nurses who had experiences in feeding children with CLP, irrespective of whether they had used the Long Nipple), as well as 7 participants from the general public (2 housewives, 2 prospective graduating nursing students who had completed pediatric nursing training, and 3 general college students without pediatric nursing training). The housewives and students were not required to have experience in feeding children with CLP. As this was a pilot study, the sample size was not determined in advance, and the effect size was estimated to measure the power of detection with these seven participants.

Adhering to the inclusion criteria for the comparison group, we recruited four nurses who mastered the Kumagai method. The university hospital in Japan where the nurses worked provides top-level treatment for children with CLP. It has a specialized CLP center that performs approximately 400 CLP-related surgeries annually. It also provides approximately 100 cases of support services yearly to neighboring hospitals upon request for special help for a child with feeding difficulties. Ms. Kumagai independently trains the nurses in the Kumagai method in this hospital. She certifies one as a master by observing how the nurses actually feed the children. She selected and invited certified nurses to participate in this study. The researchers explained the study to them and compared their actions with those of other participants.

### 2.3. Setting

This research protocol was registered with the University Hospital Medical Information Network (receipt number: UMIN 000049549) [18]. The data were collected individually for each participant. A camera was mounted on a 140 cm tripod in an open, pressure-free space, and a standing position marker was placed 220 cm from the tripod. The participants were asked to stand at the marker and perform the feeding gesture on an infant lactation practice doll named Taakun, which was designed to resemble the shape of a newborn baby (approximately 50 cm in height and 2800 g in weight). The doll’s head is made of silicon, and its lips and palate were cut using a cutter to resemble an infant with left unilateral CLP. The feeding bottle used was an empty Long Nipple. The camera mode was set to 1920 × 1080 pixels at 30 frames per second to capture videos of the frontal view of the participants. In addition, a wearable camera was attached to each participant’s ear to capture pseudo-viewpoint videos. The wearable camera mode was set to 1920 × 1080 pixels at 60 frames per second.

The participants performed the actions according to the following questions: “What feeding pose do you adopt when feeding the child?”; “How do you hold the bottle?”; “How do you insert the nipple into the mouth?”; “Where do you place the nipple to continue feeding?”; and “How do you remove the nipple?” These five questions are based on the main technical items in the Kumagai Method [13]. The participants did not know the questions in advance, nor had they the time to practice, and were asked to act as they saw fit. Then, the intervention was applied, and the participants were asked the same questions. However, they were told that they could choose to perform actions similar to or different from that of the first step and that they should act in the most appropriate way for feeding the infant doll with CLP. The comparison group was also asked the same questions to perform the Kumagai method.

### 2.4. Intervention

After the participants performed the first set of actions, we showed them a 2.5-min YouTube video explaining how to feed infants with CLP using the Kumagai method [19]. The video was created independently by Ms. Kumagai, and animation was used to explain the important steps that can be mistaken, such as the structure of the Long Nipple, how to insert it (angle, speed, and timing of insertion), and how to maintain feeding (timing of pinching the nipple). The videos were condensed into a short time frame in favor of familiarity, without explaining all items. Items marked with “#” in Table 1 are explained in the video.

### 2.5. Measures

A 24-item checklist (Table 1) was created to evaluate each feeding action of the Kumagai method [13]. Some procedures, such as adjusting the force of pinching the nipple according to the child’s sucking ability, or responding to changes according to the child’s movements, were not evaluated because they could not be reproduced using the doll. For each item, the validity of the participant’s action according to the Kumagai method was evaluated as yes or no. The assessors were two Kumagai method specialists (Ms. Kumagai and Ms. Hirai, a vice chief director of the same department), who independently viewed each participant’s videos (frontal view and viewpoint) and evaluated them. The assessors were unaware of the participants’ backgrounds, and their faces in the videos were mosaicked. They were also unaware of whether the videos were pre- or post-intervention. To evaluate the inter-rater reliability, we calculated the Cohen’s Kappa coefficient between the two assessors. The item-rated discrepancy was discussed with the researchers, including the two evaluators, until a consensus was reached.

**Figure 1:**
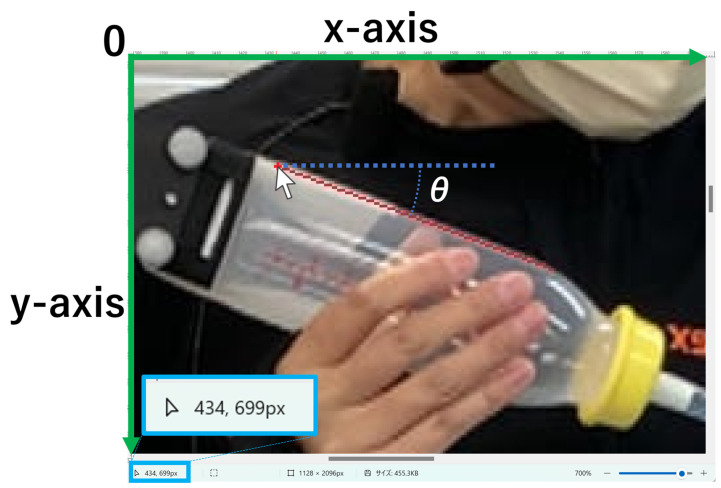
Calculating the angles using Microsoft Paint. Note: A straight line was drawn on the side of the bottle. The cursor was placed on the edge of the line, and the number of pixels was displayed. In this figure, the x-coordinate = 434 and y-coordinate = 699. The lower-right ends of this line were x = 538 and y = 738. Therefore, tangent θ = (699 − 738)/(538 − 434) = −0.38; accordingly, θ = −20.56°. The bottle angle was determined by subtracting the angle of the straight line on the ceiling from this angle.

In the 24 evaluation items, nos. 11 and 16 were related to the angles [13]; however, it is difficult to visually evaluate the angles in the video. Therefore, the following were evaluated from the paused video images: (i) whether the bottle was horizontal when the nipple touched the lip and (ii) whether the angle of the bottle changed as the nipple entered the doll’s oral cavity. The images were saved when the nipple touched the lip (“moment of lip”) and when the nipple was placed in the oral cavity and held still (“moment of mouth”). The images were opened in Microsoft Paint (Microsoft, Redmond, WA, USA), and the side of the bottle was drawn in a straight line. The slope of the straight line was calculated by confirming the 2-point number of pixels at the ends of the line on the XY coordinate, and the angle was calculated using the tangent (Figure 1). To accommodate the slight tilt of the camera during recording, a straight line was drawn in the same manner on the border of the ceiling, as shown in Figure 1. The bottle angle was corrected using the tilt of the straight ceiling line. To evaluate (i) and (ii), the “moment of lip” angle and the difference between “moment of lip” and “moment of mouth” angles were obtained, respectively.

### 2.6. Analysis

The feeding actions of the participants pre- and post-intervention were evaluated by the two assessors using the 24-item checklist. The inter-rater reliability was assessed by Cohen’s Kappa coefficient. The strength of agreement was defined as follows: 0.41–0.60 = moderate; 0.61–0.80 = substantial; and 0.81–1.0 = almost perfect [20]. The effect of the intervention was examined using the McNemar test (Yates’ correction) for the number of “yes” before and after the intervention. The 24 items were divided into 8 explained (no. 7–10, 12, 14, 16, and 18) and 16 unexplained (no. 1–6, 11, 13, 15, 17, and 19–24) items in the video, and each was used for the McNemar test. Subsequently, the Breslow–Day test was performed for subgroup analysis. Moreover, chi-square tests were used for differences between explained and unexplained items in the number of “yes” responses post-intervention.

The angle of the feeding bottle was compared using the Kruskal–Wallis test for the three groups—four Kumagai method specialists and participants before and after the intervention. Dunnett’s test was performed for significant differences as an a posteriori test. The test compares between groups. The comparison group’s movements were compared to those of the participants before and after the intervention. John’s Macintosh Pro (JMP; SAS Institute Inc., Cary, NC, USA) was used for statistical analysis, and the significance level was set at *p* < 0.05.

### 2.7. Ethical Consideration

This study was approved by the Institutional Ethics Review Committee of Kyushu University (22136-01) and was performed in line with the principles of the Declaration of Helsinki and its later amendments. Participants and the comparison group were informed that they could freely choose to participate and could withdraw their consent at any time without any disadvantages. Each participant’s face was mosaicked while evaluating their feeding actions. The video data were stored in password-controlled memory to prevent data leakage. Written consent was obtained from all participants after they had agreed to participate. Participants were paid an honorarium of JPY 1000 (approximately USD 7) per hour.

## 3. Results

Two midwives (A, B), pediatric nurses (C, D), housewives (E, F), prospective graduating nursing students (G, H) with pediatric nursing training, and three general college students (I, J, and K) without training participated in this study, and none withdrew. All were women, and their heights were <150 cm (*n* = 1), 151–160 cm (*n* = 6), and >161 cm (*n* = 4; range: 147–168 cm). Only one midwife (B) had experienced a live demonstration of the Kumagai method by a specialist at the hospital where she worked. None had watched a YouTube video of the Kumagai method. The nursing experience of the comparison group ranged from 18 to 36 years.

The Kappa coefficient was 93.0% (*p* < 0.001). Table 1 shows the number of participants meeting the assessment criteria. The number of “yes” increased for “holding the bottle” (four items) post-intervention, although for less than half of the participants. Responses to other items remained almost the same. Items 11, 12, 14, 15, 18, and 21 were rated “yes” for only two participants in post-intervention.

Table 2 shows the number of “yes” before and after intervention in all items, categorized by the explained and unexplained items. The analysis of the total number of “yes” using the McNemar test revealed a significant increase (χ-squared = 14.02, *p* < 0.001). The paired odds ratio was 45/15 = 3, and the power of detection was 0.97, proving that the number of participants was sufficient. McNemar’s test showed a significant difference in the explained items (χ-squared = 11.12, *p* < 0.001) but not in the unexplained ones (χ-squared = 3.56, *p* = 0.059). The Breslow–Day test revealed a significant difference (χ-squared = 4.58, *p* = 0.032), indicating significantly different “yes” evaluations before and after intervention between explained and unexplained items. In addition, the number of “yes” and “no” for the explained items post-intervention was 28 (6+22) and 60 (4+56), respectively, and for unexplained items, 94 (71+23) and 82 (11+71), respectively. The chi-square test for these differences was significant (χ-squared = 20.48, *p* < 0.001).

**Figure 2:**
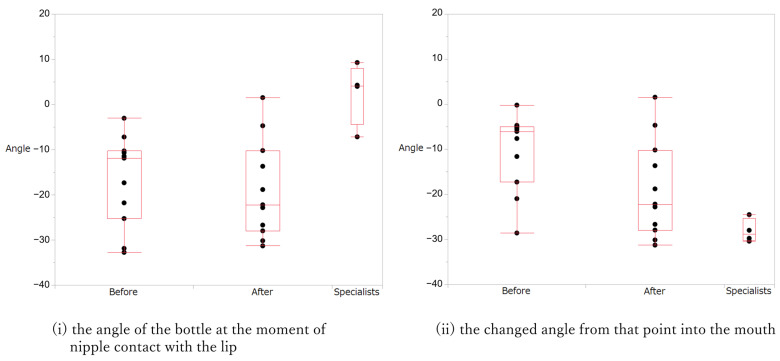
Differences in the actions of the participants in the first and second instances and those of the specialists.

Figure 2 shows the distribution of (i) the angle of the bottle when the nipple touched the lip and (ii) the change in angle from that point to the mouth. At time (i), the Kumagai method specialists positioned the bottle almost horizontally, whereas most participants maintained a negative angle during both times. The actions of the four specialists were significantly different from those of the participants (*p* = 0.013). Dunnett’s test showed a significant difference in the participants’ actions at both times and specialists’ actions (participants pre-intervention vs. specialists: *p* = 0.006; participants post-intervention vs. specialists: *p* = 0.003). The change in the angle at time (ii) was distributed around −30° for the specialists, whereas that for the participants had various angles in the first evaluation and around −10° in the second evaluation. The Kruskal–Wallis test showed a significant difference between the three groups (*p* = 0.016). Dunnett’s test showed a significant difference between participants at each time point and specialists (participants pre-intervention vs. specialists: *p* < 0.001; participants post-intervention vs. specialists: *p* < 0.001).

## 4. Discussion

We examined the effectiveness of watching an instructional video on improving the technique to handle the Long Nipple. Despite the short duration of the video, the participants’ actions significantly improved after viewing it and were closer to the Kumagai method. In particular, the explained techniques significantly improved the acquisition rate compared to the unexplained ones. Short videos are easy to watch, easily accepted, and effective in improving the skills of first-time viewers. Our instructional video was made with animations that clearly show the oral cavity. This may have contributed to a better understanding of the participants and improved the acquisition rate. However, post-intervention, the total number of explained items acquired was significantly less than that of the unexplained items, and only half of the participants were able to acquire at least one. Moreover, the bottle angles at the two moments were significantly different between the participants and the specialists. Furthermore, the actions according to the Kumagai method could not be completely acquired by watching only the short video.

A previous study [13] showed the procedures “Move the bottle horizontally and lightly place the nipple against the lower lip” and “Tilt the nipple (in contact with the tongue surface) about 10 degrees so that it fills milk”. It recruited five Kumagai method specialists, including Ms. Kumagai, and collected and analyzed their data. However, it might be difficult for humans to accurately determine the angles in space. In the present study, the actual angles evaluated objectively were approximately horizontal when the nipple touched the lip and changed to approximately 30°, not 10°, as the nipple entered the child’s oral cavity. In contrast, the angles of the participants were already tilted negatively when the nipple touched the child’s lips and even more negatively when it was inserted into the oral cavity. This would cause the milk to splash onto the child’s face if the bottle contained milk. Moreover, when the nipple is inserted into the mouth, the milk automatically enters the mouth, causing the child to choke. The observance of the angle is certainly important; however, the challenge of accurately implementing the degrees persists. Not only is being conscious about the angle of tilt of the bottle an important factor but also assessing the appropriateness of the angle based on whether milk would spill when moving the bottle, whether the nipple would touch the child’s tongue surface, and whether tilt would fill the nipple with milk are important as well.

Placing the nipple against the lower lip informs the child about the initiation of feeding and aims to promote their sucking reflex by smoothly inserting the nipple [13]. Furthermore, the nipple is inserted into a depth of 2–3 mm, where the infant can manipulate the nipple with their tongue most effectively [13]. Although these steps are explained in the video, the underlying reasons are not. Consequently, the participants may not have understood why it was necessary and may have performed it in their preferred manner post-intervention. However, mastering these actions takes time, even after understanding the rationale, thus highlighting the need for direct instruction from specialists.

In the present study, the inter-rater reliability of the two evaluators was 93%, indicating a high level of similarity between their viewpoints. Although the Kumagai method is new with only a few specialists, the technique has proved to be clearly established. A recent study examining the appropriateness of the content of videos on feeding instructions for children with CLP publicized as YouTube videos found no videos with excellent content and only one-third with optimal content [21]. Our video introduces a proven technique that has been established by specialists and contributes to improving technique acquisition.

### 4.1. Limitations

The reliability of the independent evaluators was high. However, assessor bias may have been introduced as the assessment was subjective after watching the video. To further complement the visual evaluation, we added two objective evaluations of the angle of the bottle using images. The angles evaluated in the two scenes were collected as two-dimensional static images from the frontal view. Moreover, the participants’ heights were not considered, and the videos were recorded from a fixed position, introducing the possibility of small errors in the actual angles in a three-dimensional space.

### 4.2. Further Research

This study has shown that the video is effective for technique acquisition. In the future, more participants should be included to confirm its effectiveness, and a full video explaining all the techniques should be created.

This study setting lacked real-world conditions owing to the use of a doll. Infants may resist, arch their backs, or spit the milk out. Although certain environmental conditions need to be limited, addressing how to handle them is clinically important. Therefore, it is necessary to clarify how Kumagai method specialists deal with real infant movements, such as resistance to feeding.

## 5. Conclusions

We revealed that watching a video demonstrating the Kumagai method significantly contributed to learning the use of the Long Nipple. We also clarified the significant differences in the feeding actions of the participants and specialists. To learn the Kumagai method completely, it may be necessary to explain the rationale for each movement in video and to supplement direct instruction. In addition, it is important to deal with actual children with feeding difficulties when the child refuses to be fed, and future research should clarify how the Kumagai method specialists deal with them.

## Figures and Tables

**Table 1:** Evaluations of participants’ actions before and after watching the instructional video (*n* = 11).

No.	Item	Before	After
**Basic posture**			
1	The child’s nuchal region was placed close to the elbow joint.	6	7
2	The child was pulled close to the breast.	6	7
3	The child’s arm was held under the armpit.	3	3
4	The child’s body was close to the body.	10	9
5	The child was held in a slightly upright position.	6	8
6	The child’s body was held straight.	6	7
**Holding the bottle**			
7 ^#^	The bottle was held by the thumb, ring finger, and little finger.	1	5
8 ^#^	The nipple was pinched between the terminal joints of the index and middle fingers.	1	3
9 ^#^	The center of the thick part of the nipple was pinched.	0	5
10 ^#^	Pinching fingers were bent slightly.	1	5
**Insertion of the nipple into the mouth**			
11	The bottle was moved horizontally.	3	2
12 ^#^	The nipple was touched lightly against the lower lip before inserting into the mouth.	1	2
13	Elbow was not raised when moving the bottle.	11	9
14 ^#^	The nipple adhered smoothly from the lower lip to the tongue when inserted into the mouth.	0	2
15	The angle of insertion was controlled by the wrist.	4	2
16 ^#^	The nipple was kept in contact with the tongue surface by tilting the nipple at about 10 degrees.	3	4
17	The nipple was placed on the center of the tongue.	6	8
18 ^#^	The nipple was inserted approximately 2–3 mm from the nipple thickening at the child’s lip.	3	2
19	The nipple was kept straight when inserting it into the mouth.	4	7
20	The nipple was kept straight when pinching it.	1	5
**While feeding**			
21	The index and middle fingers were kept on the nipple when they were not pinching.	0	1
22	The nipple was kept in a fixed position.	2	3
**End of feeding**			
23	The nipple was pulled straight out at the angle of the oral cavity.	7	8
24	The nipple was turned up immediately after pulling it out.	7	8

^#^: Items explained in the video.

**Table 2:** Contingency table by total items based on the explained and unexplained items in the video before and after intervention.

		Total (24 Items)		Explained (8 Items)		Unexplained (16 Items)	
		Before Intervention		Before Intervention		Before Intervention	
		Yes	No	Yes	No	Yes	No
After intervention	Yes	77	45	6	22	71	23
	No	15	127	4	56	11	71

Note: 24 items were assessed per participant. Therefore, the total number of items was 264.

## Data Availability

The data presented in this study are available upon reasonable request from the corresponding author due to privacy and anonymity considerations.
